# What Does It Mean to “Age Well” Among British and Javanese Older Adults? A Cross-Cultural Qualitative Study

**DOI:** 10.1093/geronb/gbae085

**Published:** 2024-05-18

**Authors:** Santi Sulandari, Rachel O Coats, Taufik Taufik, Judith Johnson

**Affiliations:** School of Psychology, University of Leeds, Leeds, West Yorkshire, UK; Faculty of Psychology, Universitas Muhammadiyah Surakarta, Surakarta, Central Java, Indonesia; School of Psychology, University of Leeds, Leeds, West Yorkshire, UK; Faculty of Psychology, Universitas Muhammadiyah Surakarta, Surakarta, Central Java, Indonesia; School of Psychology, University of Leeds, Leeds, West Yorkshire, UK; Bradford Institute for Health Research, Bradford Royal Infirmary, Bradford, West Yorkshire, UK; (Social Sciences Section)

**Keywords:** Age friendly, Cross-culture, Environment, Positive aging, Successful aging

## Abstract

**Objectives:**

This study aimed to investigate perceptions of what it means to “age well” and to explore similarities and differences between a Western and non-Western culture (Britain and Java).

**Methods:**

Qualitative interviews explored how Javanese and British older adults defined aging well, establishing the similarities and differences between cultures. Javanese (*n* = 14) and British (*n* = 15) adults aged 61–80 (mean age = 68) participated. The data were analyzed using reflexive thematic analysis and organized with NVivo.

**Results:**

Four themes were identified that captured what it means to age well across cultures: (a) *good health is a springboard for aging well*; (b) *holding a positive outlook is a decisive factor in aging well*; (c) *“having enough” and “feeling safe” provide peace of mind*; and (d) *spirituality and religiosity provide tranquility.* Although both cultures mentioned similar factors, there were variations in the interpretations and emphasis within themes. For example, Javanese participants emphasized the importance of the social environment whereas British participants highlighted the physical environment.

**Discussion:**

Differences between cultures are important for understanding how best to support people as they age. For example, in Java, aging well may be best supported by providing a vibrant social environment. For people in Britain, having a safe and secure physical environment may be more important.

The global percentage of people who are 65 or older is expected to increase from 10% in 2022 to 16% in 2050 (United Nations Department of Economic and Social Affairs, 2022). Aging populations can create societal opportunities. For example, older adults can share wisdom, knowledge, and skills with younger generations, which can be culturally and practically enriching. A larger number of older adults in a population can also lead to innovations designed to meet the needs of older adults (National Academies of Sciences & Medicine, 2019). Moreover, the community can benefit from the involvement of older adults in volunteer roles within health and social services, for which there is increasing demand (Calvo-Sotomayor & Atutxa, 2022; Healy, 2004). However, due to higher healthcare needs in older age groups and a likelihood of lower financial contributions, aging populations can create economic pressures on societies (Bloom et al., 2015; United Nations, 2019). A study across Organization for Economic Cooperation and Development (OECD) countries and Brazil, Russia, India, China, and South Africa (BRICS) found that in 2010, 40% of healthcare expenditures were for those over 65 and projected that by 2060, 60% of spending will target this group (de la Maisonneuve & Martins, 2014). Additionally, the projected impact of aging pressures on the public debt burden is estimated to be approximately 180% of gross domestic product (GDP) in G20 advanced economies and 130% of GDP in G20 emerging economies within the next three decades. To maintain the current levels of public debt-to-GDP ratios, G20 countries would need to augment tax revenue by approximately 4.5%–11.5% of GDP by 2060 (Rouzet et al., 2019).

Due to these projected aging-related pressures on health systems, there has been a growing interest in understanding how adults can maintain personal well-being in later life. In this present study, we focused specifically on the concept of “aging well.” This differs from other theoretical frameworks that have also been used in the field, such as (a) “successful aging” (Rowe & Kahn, 1987, 1997), which has a biomedical focus and is limited to avoiding disease and disability; maintaining high cognitive/mental/physical function; actively participating in life; and adjusting psychologically in later life (Kim & Park, 2017) and (b) “healthy aging,” which is limited to maintaining health and optimizing functional ability in later life (Haveman-Nies et al., 2003; World Health Organization, 2021). We selected the concept of “aging well” due to its more nuanced approach that focuses on overall well-being in later life although taking into account the dynamic nature of aging, adaptation, and meaning-making. This is distinct from the concepts of successful aging and healthy aging due to the focus on the lived experience of individuals, rather than objective, physical outcome indicators. Fernández-Ballesteros et al. (2013) suggested that the “aging well” literature is a new paradigm in the field of aging that emphasizes healthy, successful, active, optimal, competent, vital, and productive aging, and is a universally recognized term adopted across international contexts, making it appropriate for use in cross-cultural studies.

Directly measuring “aging well” in quantitative designs has been rare; instead, most studies utilize proxy concepts, such as “well-being,” “quality of life,” and “life satisfaction” to capture this concept (Brett et al., 2019; Carandang et al., 2020; Fastame et al., 2021; LaCroix et al., 2016) as it is quite broad and hard to encapsulate using a single measure. Qualitative research may be better suited for understanding this nuanced concept due to the in-depth investigation entailed and its potential for generating rich insights (Mays & Pope, 2000). Even though there is some debate about the efficacy of qualitative approaches in research (Mays & Pope, 2000), de Medeiros (2023) suggested that instead of comparing it to quantitative research, it is crucial to begin with defining the research question, and identifying which approaches and specific methods/techniques will provide the most effective way of answering it. Qualitative research provides a unique chance to advance theory, elevate underrepresented groups’ perspectives, and investigate the complexities of people’s daily lives. This holistic approach generates vivid narratives allowing readers to empathetically gain insight into everyday activities and social interactions, and enabling researchers to gain a more in-depth understanding of the issues facing older people (Warren-Findlow, 2013).

A relatively recent qualitative study in Sweden (Halaweh et al., 2018) identified that aging well was influenced by several factors, including feelings of joy, maintaining independence, achieving financial security, active social engagement, and good health. These insights would have been challenging to generate using deductive and restrictive quantitative methods. However, most qualitative studies on this topic come from Western countries and there is a lack of studies from non-Western nations (Conkova & Lindenberg, 2020; Lucchese et al., 2022; Neville et al., 2020; Waugh & Mackenzie, 2011). Furthermore, none compare Western and non-Western perspectives. This is important because definitions of what it means to age well are likely to differ between cultures. There has been one study (Nguyen & Seal, 2014) that interviewed older participants from two ethnicities (Chinese and Hmong) in the United States, but both groups were from an Asian background, relocated to a single Western country, and as such it was not a direct Eastern–Western cross-cultural study.

Of the qualitative studies that have been conducted on aging well in non-Western groups (Yoon et al., 2020; Zanjari et al., 2016), none of them have recruited participants from Indonesia, or Indonesia’s largest ethnic group, the Javanese. Although qualitative studies into other aspects of aging have been conducted in Javanese groups (Soetjiningsih et al., 2019; Sulandari et al., 2017; Taufik et al., 2023), none have focused on aging well. For example, Soetjiningsih et al. (2019) focused on exploring successful aging and Taufik et al. (2023) focused on quality life. In the UK, one qualitative study (Ylänne et al., 2010) into “aging well” has been conducted; however, this only assessed a variety of health and well-being messages in advertising. The data were collected from British magazine advertisements, which may lead to limitations in data comprehension due to its secondary nature compared with interpretation derived from direct interaction with participants.

With an estimated population of around 100 million individuals, the Javanese people constitute the largest ethnic group in Indonesia and Southeast Asia overall (Indonesian Central Bureau of Statistics, 2010). There are several reasons to suggest that different ethnic groups will have different experiences of aging well. For example, most older Javanese are Muslim and live in an extended family across several generations, which is not the norm in Western cultures. This environmental difference (such as extra support) might influence their views on life in old age. Findings from research in this group could also have relevance for other similar cultures, such as populations in Pakistan, Bangladesh, Saudi Arabia, and Malaysia, where there is also a lack of research into aging well. In comparison, the UK has a population of about 67 million individuals (Office for National Statistics, 2022) and is representative of Western culture in various ways. For example, the UK has an individualistic culture and is fairly ethnically diverse, similar to other Western nations. It provides a range of government-funded programs and initiatives to support healthy aging and community engagement, which frequently encompass healthcare services, social care, pension schemes, and community participation programs (Department of Health, 2001; Government Office for Science, 2016; Office for Health Improvement & Disparities, 2022; Woods & Crampin, 2020). In comparison, Javanese culture is collectivist and emphasizes family values. The government may enact initiatives to promote the healthcare requirements and social well-being of older individuals, but social and familial networks frequently play a vital role in providing assistance to older people in Indonesia.

Due to these kinds of variations, experiences of aging are likely to vary between cultures, and might result in diverse perspectives on what it means to “age well.” It is important that we determine the nature of these variations to inform best practices, for example, when it comes to planning culturally sensitive interventions. The objective of the current study was therefore to investigate what it means to “age well” and to explore similarities and differences between cultures (Javanese and British). Understanding this could inform interventions to support adults with aging well and ensure that such interventions reflect cultural differences.

## Method

A qualitative research design was used. A semistructured interview schedule was developed, consisting of open-ended questions pertaining to the study aims.

### Ethics

This study was reviewed by the University of Leeds, School of Psychology Research Ethics Committee (approval number: PSYC-450, approval date: 29/01/2022).

### Participant and Recruitment

Purposive sampling was used to recruit participants aged 60+ from two different cultures (Javanese and British). Javanese older adults were recruited via social media, with posters and accompanying text distributed on Instagram, Facebook, and WhatsApp stories. Younger people who saw the adverts were asked to pass on the information to their older relatives/friends. Anyone who wanted to take part contacted the researchers. British older adults were recruited from the School of Psychology, University of Leeds (UK) Successful Aging Panel via emails. Interested participants contacted the researchers. Social media was also used, and posts were shared on Facebook. Prospective participants were given the opportunity to ask questions and were asked to provide recorded verbal informed consent prior to participating.

### Data Collection

Participants were invited to participate in qualitative semistructured interviews exploring their views on what it means to age well. A semistructured interview guide was designed to capture the distinctive experiences and perspectives of individual participants (see Supplementary Section A). S.S. conducted all interviews from March to August 2022. Interviews with Javanese participants based in Indonesia were conducted in Indonesian mixed Javanese language whereas interviews with British participants based in the UK were in English. Participants reported demographic information prior to the interview see Supplementary Section B. The interviews were conducted remotely via telephone (*n* = 3) or video platform (*n* = 26) and were audio or video recorded and then saved to a secure cloud server (Onedrive), depending on the participants’ preferences. The interviews lasted from 23 to 80 min (mean = 40 min).

### Data Analysis

The data were analyzed using reflexive thematic analysis (Braun & Clarke, 2006, 2019). This method uncovers themes and patterns of significance across the data set. The analysis begins with data familiarization, followed by comprehensive data coding, topic seeking, theme evaluation and revision, and theme definition and labeling. Throughout the analysis, an inductive method was predominantly used. Complete coding was used, and data were coded according to both semantic (descriptive), and latent (interpretive) meanings. An example of a semantically coded quote was “I don’t like to bother people, so it’s best to try and keep healthy,” which we coded as “do not want to be a burden on anyone else because of poor health.” On the other hand, a quote like “*Allah* teaches us to be patient. You have to be patient and *tawakkal* (put all of your effort into doing something, but then again, be patient). So that we can have peace and no trouble in our lives” was identified as a latent code, which we coded as “patience is a value in their religious belief system which helped them to age well.” Related codes were grouped together under central organizing principles, which were then transformed into candidate themes. Candidate themes were compared with coded data extracts and the data set as a whole, and revisions were made as needed. Interviews were transcribed and coded by S.S., R.C. and J.J. reviewed *n* = 6 transcripts (20%) to enable triangulation of the data analysis. Three of these were Indonesian transcripts which were translated and coded by S.S initially. T.T., an Indonesian researcher who has experience in conducting qualitative research and is fluent in both Indonesian and Javanese language, also checked all Indonesian/Javanese transcripts and coding to further enhance triangulation of the analysis. Any discrepancies were resolved through a consensus discussion. An example of disagreement between authors is provided in Supplementary Section C. Although the coded and initial themes were developed by the first author, theme development, review, and revision took place with consultation from all coauthors. To increase rigor and dependability, the first author kept a self-reflective journal/diary throughout the research, listing, among other things, her assumptions on what aging well means and any significant thoughts throughout the data collecting or analytical procedure. All codes and themes were unpacked, sorted, and organized using NVivo Release 1.7.1 (released in March 2020).

## Results

Participants in this study were 29 older people from both countries (4 British men and 11 British women; 7 Javanese men and 7 Javanese women; see Table 1). The Javanese participants ranged from 61 to 80 years old (mean = 67) and British participants were 63 to 78 (mean = 69). Seven of 14 the Javanese and 7 of 15 the British had a higher education degree.

**Table 1.** Characteristics of the Participants

Javanese	*n*	British	*n*
Health condition		Health condition	
Have health issue(s)	5	Have health issue (s)	8
Healthy	9	Healthy	7
Living arrangement		Living arrangement	
Live alone	2	Live alone	6
Live with spouse	3	Live with spouse	8
Live with daughter/son	2	Live with a husband and a daughter	1
Live with extended family	7		
Marital status		Marital status	
Married	8	Married	9
Widowed	6	Widowed	4
		Divorced	1
		Single	1

The thematic analysis identified four themes that are described later (see Supplementary Sections D and E for additional information). Quotes are followed by a code denoting which participants said them; for example, B for British, J for Javanese, and then a unique number.

### Theme 1: Good Health Is a Springboard for Aging Well

Participants emphasized that maintaining good health was crucial to enabling their active engagement in physical, social, religious, and personal activities. Older people felt good health could unlock an extensive range of opportunities for personal fulfillment, social connections, and continued growth in their later years. Social connection in particular was critical to aging well, and good health enabled this. With good health, older people could meet outside with friends and family, volunteer, help others in need, attend religious/community services, participate in social gatherings, and spend time together in the community. This view was shared similarly in both cultures:

Keeping yourself fit and healthy is important because if you’re full of ailments, you can’t go out and meet people, and unfortunately, people get fed up with you moaning about your health. (B4)

If people are healthy, it means they can worship in peace, right? People will not be able to worship when they have health problems, so being healthy is important. Being healthy means being able to enjoy everything. For example, if I have an event or gathering invitation, I will be able to attend when I am healthy, but if I am sick, I will not be able to make it. Usually, after the *Fajr* prayer, I do recite, but yesterday I caught a cold, so it is not possible to recite the *Quran* for the following 2 to 3 days. So, with God’s willing, if you are healthy, you will definitely be able to do the religious practices. Being in good health also allows me to go for a walk in the morning. (J1)

Participants from both cultures noted that to stay healthy they had to have a good lifestyle, such as: exercising, consuming healthy foods, avoiding smoking and alcohol, managing time between activity and rest, and praying and meditating:

When you are in good health, you can enjoy life. That is aging well ... It is still important to take care of your health … being able to take care of it through a proper lifestyle and a positive mindset, as well as having a balance between activities and rest. (J2)

Always keep being healthy by eating well, doing exercise, keeping busy, making sure that any ailments that come up are dealt with, and taking the appropriate food supplements. Going to the shops, surrounding area, or church, by walking. So, I do quite a lot of walking and Pilates. (B6)

Participants from both cultures highlighted the effect of the COVID-19 pandemic in preventing them from being outside or interacting with people. Adjusting to postpandemic conditions, one British participant mentioned:

I initially found the pandemic very difficult because I wasn’t seeing friends and family face to face, but I’ve gotten used to it now. I’ve lost a bit of confidence about making a lot of face-to-face contact with people. I’m going to try and get that confidence back up again. So, I actually need to try and venture out more and meet people face-to-face more. (B5)

A further reason given for the need to be healthy was because it affords independence. Participants from both cultures did not want to be a burden on anyone else, especially their families:

If you have to rely on other people to come and pick you up, all the rest of it becomes a bit of a pain for people. I don’t like to bother people, so it’s best to try and keep healthy. (B7)

I thank God. I was accompanied by health from *Allah*, so I don’t bother my children, grandchildren, or neighbors. I think that’s good. (J6)

Participants’ conceptualization of health was broad, including physical, psychological, and cognitive health. Some participants noted that having mental health concerns, such as depression or cognitive decline, was a barrier to aging well. Both British and Javanese participants were aware of the importance of psychological health, but the British spoke more often about this than the Javanese. One British participant mentioned that mental health could prevent engagement in activities and reduce motivation.

If people become depressed, it’s difficult to get motivated to do things. (B2)

Interestingly, only British participants referred to the importance of cognitive health for aging well (see Supplementary Section F, Figure 3A):

I think if you’re aging well, mentally bright, and able to communicate, then that’s a nice thing to be able to do that a lot of people can’t do most of the time because of dementia. I find that a lot of people forget things and don’t behave the way they would normally. If I had a mental health issue or dementia, then there’s no way that I could work on my films and still edit them and be creative. I do a lot of writing as well, so I’m very pleased that my brain still functions normally. (B8)

In summary, health, both physical and psychological, was considered a prominent factor in aging well in both countries that helped the individuals to enjoy life with a wide array of opportunities for personal satisfaction, social relationships, and ongoing development during their later stages of life.

### Theme 2: Holding a Positive Outlook Is a Decisive Factor in Aging Well

Holding a positive outlook facilitated meaning and purpose in life, which led to enjoying life, being satisfied with life, being happy, and being content. The positive outlook described by participants included accepting health conditions, not complaining about life circumstances, understanding individual differences, being open-minded, being optimistic, and having a positive attitude toward aging. Even though participants emphasized the benefits of health, living close to family, and connecting with others, holding a positive outlook was described as compensating when these preferred life situations were not present. This view was shared similarly in both cultures:

People who are aging well are those who are more positive and are not denying the difficulties they’ve had in their lives. They tend to have a more positive attitude, not being judgmental of other age groups or ethnicities, accepting other people, and not complaining all the time. (B5)

Aging well is an optimistic attitude; keep going, don’t give up, don’t get tired, and keep the spirit up. (J12)

Some participants from both cultures also stressed the importance of personality to help them age well, such as patience, resilience, friendliness, reflectiveness, determination, sense of humor, confidence, and cheerfulness. Two of them mentioned:

Yeah, personality helps to age well. If we’re not friendly and don’t communicate with people, it’s difficult. You know, to get that conversation going, some people are not quite as easy to talk to, and it can be quite difficult. (B2)

A person who ages well is patient and loving at heart. They are becoming older but still very persistent. Despite facing several challenges, they never give up. (J4)

Some Javanese participants identified patience as a value in their religious belief system which helped them to age well. In comparison, the British participants stressed the benefits of being outgoing:

*Allah* teaches us to be patient. You have to be patient and *tawakkal* (put all of your effort into doing something, but then again, be patient). So that we can have peace and no trouble in our lives. Right, if you are patient, there will be no problem. (J9)

I would say aging well means being outgoing, being the sort of open and friendly person without bringing their own problems to any relationship, and being good at communicating. (B12)

Interestingly, although the beneficial personality factors identified varied between cultures, both had the same purpose: helping to build and maintain good relationships with others:

People who are aging well are very kind and thoughtful. I’m very friendly and can talk [nicely] to people. So, I think that helps me age well. (B2)

Being patient [helps me] to avoid conflict and understand others’ circumstances. (J10)

In summary, participants identified that patience, being outgoing, and having a positive outlook helped them to have more purpose in life and build good relationships with others which in turn allowed them to age well.

### Theme 3: “Having Enough” and “Feeling Safe” Provide Peace of Mind

Participants from both cultures identified finances and the environment as important factors in contributing to aging well. Although the British participants highlighted that having good financial resources was important to aging well, the Javanese people identified the fact that a willingness to adjust to various financial and living circumstances was the key. They were focused on being grateful for “having enough” and avoiding making complaints about wanting more:

If they have financial problems, I think someone is going to be worried all the time about money; like at the moment, they can’t imagine how they’re going to cope with their heating bills. That will make people anxious; it will affect their mental health. (B9)

From my point of view, aging well for older people means feeling like you have enough. For example, my house is just simple, but I feel that’s enough, and I am grateful. (J5)

Some older individuals from Java emphasized that financial matters frequently served as a crucial catalyst for interpersonal conflicts within families or among neighbors, inevitably impacting their relationships:

It’s simply an economic problem. If economic stability isn’t there, some people tend to get upset easily, argue with their children and grandchildren, and so on. (J10)

Moreover, the importance of being physically and socially safe was highlighted by participants from both cultures. British participants put more emphasis on the physical environment: they preferred to live in a house that matched their capacity and preference (fewer stairs, close to amenities, and having a garden or being close to nature). Some British participants also noted that aging well involved not living in a care home:

A nursing home is a place to stay when you are not very well. So, a person who is aging well is very independent. Even though they might not be physically well, they will try to find ways to live their lives independently. (B15)

On the other hand, Javanese participants placed less emphasis on the physical environment and were more focused on the importance of their social surroundings, especially the significance of family presence. They highlighted the value of living close to family because they were available to provide support and share in experiences:

All my three children are already married. One of them lives a bit far away with her husband, but my two sons live either side of me: my house is right in the middle. In accordance with what I dreamt of, I feel comfortable in my old age because my children and grandchildren are around me. (J2)

Additionally, although the Javanese participants placed more value on social relationships compared with the British participants, both groups acknowledged that having social support provided warmth. Interestingly, older individuals from British backgrounds tended to place a greater emphasis on the importance of friends, rather than focusing on their family dynamics:

We don’t have family close by. [But it is helpful to be] with friends and have a nice chat together and talk about something interesting; talk about problems. If they’ve got some difficulty, they feel they’re able to ask for help. (B14)

I keep in touch with my friends by, for example, having lunch with the girls and occasionally going out. So, I mostly go for dinner; but sometimes one or two friends will invite me to stay in a house. (B15)

Overall, both groups of participants identified financial resources and the environment as key factors in supporting aging well, even though there were variations within how these were characterized.

### Theme 4: Spirituality and Religiosity Provide Tranquillity

Some participants from both cultures reported that faith influenced their view of old age. Some believed that being religious or conducting religious activities helped them to age well:

The aging process is just a natural part of life. I pray, meditate, and talk to God. It’s that sense that there is something much greater than me. There is a greater power that gives me a sense of meaning. (B5)

Reading the *Quran* provides peace of mind; it turns out to be true. I often read the *Quran* and feel satisfied and happy. (J12)

Spirituality and religiosity were highlighted by both British and Javanese participants as factors that helped them to find tranquillity because they were getting closer to God:

I think it’s a Christian outlook. It’s a spiritual and faith thing that supports my ability to enjoy life and to be content. In terms of our attendance at church and our reading and prayer, that is key to the way we are, I feel. The way that God has been faithful to us and given us the life that we have at the moment. (B12)

Praying is good. It turns out that the reward that can already be felt is being tranquil. Practicing the night prayer, the *dhikr*, and getting close to *Allah* is very good. Talking to *Allah* makes me feel very good. (J14)

For some, the primary driving force behind their engagement in religious practices stemmed from their conviction in the existence of an afterlife. However, Javanese participants more frequently expressed this sentiment than British participants:

Religious practices are conducted to provide for the afterlife. The next day, when you leave this world as you are getting older, what you’re looking for is having enough provision in the afterlife so that *Allah* will accept it and grant us a heaven. (J9)

Social and religious activities were undertaken with the purpose of ensuring sufficient provisions for the afterlife, but also because they provided tranquillity and peace in the here and now.

**Figure 1.**
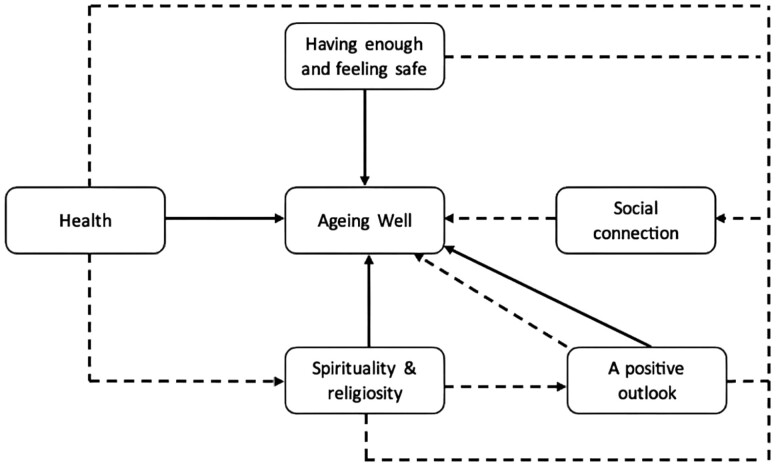
The interaction among themes regarding aging well. Good health, a positive outlook, a sense of safety, and spirituality and religiosity have the potential to enhance social connections, thereby contributing to aging well. The factors of spirituality and religion may have direct influences on aging well; however, in certain instances health may also contribute significantly to the attainment of aging well by enabling older adults to engage in religious activities. Additionally, it is worth noting that spirituality and religiosity may foster positive perspectives among older participants, and it is this that ultimately leads to aging well. Solid arrows represent a direct relationship or flow and dotted arrows denote possible indirect relationships.

Based on the available data, it is evident that there exists a potential for interaction between the various themes (see Figure 1). Good health, a positive outlook, a sense of safety, and spirituality and religiosity have the power to improve social relationships, and so contribute to the feeling of aging well. Furthermore, although most factors have direct benefits for aging well, in some cases, health may also play an important role by allowing older people to participate in religious activities and hence impacts aging well indirectly in this way. Furthermore, it is worth emphasizing that spirituality and religiosity may generate positive viewpoints among older people, which eventually contributes to aging well.

## Discussion

Four themes were identified that encapsulated what it means to older adults to age well: good health is a springboard for aging well; holding a positive outlook is a decisive factor in aging well; “having enough” and “feeling safe” provide peace of mind; and spirituality and religiosity provide tranquillity. Although the key themes for both cultures were the same, there were differences of emphasis within them. For example, in terms of good health (theme 1), British participants had more awareness of the importance of cognitive health than the Javanese. Moreover, in terms of positive outlook (theme 2), Javanese participants emphasized the importance of patience and Britons emphasized the importance of being outgoing, but both served the same ultimate goal of building and maintaining relationships. Regarding “having enough” and “feeling safe” (theme 3), the British participants highlighted the importance of financial stability, although the Javanese were more focused on being grateful for what they had. Additionally, Javanese participants were more focused on social relationships and British participants were more focused on physical environmental comforts. Lastly, both cultures acknowledged the importance of spirituality and religiosity (theme 4), but there was a greater focus on religious practices and the afterlife in the Javanese.

Our findings extend the literature in two main ways. First, they support previous research suggesting health is important to aging well (Griffith et al., 2018; Hilton et al., 2012), although also extending this previous knowledge by identifying a potential underlying mechanism in how health contributes to aging well: health is important because it affords participation in various activities, including physical exercise, social interactions, and religious practices. This is important because these activities help older people feel content and have a more meaningful life. Intriguingly, older Javanese participants reported fewer concerns regarding their psychological well-being in comparison to their British counterparts, and they did not raise any concerns pertaining to cognitive health. This occurrence may be attributed to a potential lack of awareness among the older Javanese population regarding psychological and cognitive well-being (Putri et al., 2021). For example, an Indonesian study on 1,147 individuals from a middle-aged population found that more than 67% of the participants exhibited a deficient understanding of dementia (Nugraha et al., 2022). Furthermore, this disparity may be attributed to average life expectancy, which is lower in the Javanese, and therefore fewer people experience dementia. Additionally, cultural attitudes toward a cognitive decline in older individuals, regarding it either as a natural part of aging or as a pathological condition (Winarni & Triratnawati, 2023), could also explain the discrepancy. Raising awareness in this group may be helpful in improving the identification and management of dementia.

Second, our data support findings from previous studies suggesting the physical and/or social environment affects older people’s quality of life (Garner & Holland, 2020; Tiraphat et al., 2017) and well-being (Cramm & Nieboer, 2015; MacKerron & Mourato, 2013; White et al., 2019). Our data extend existing knowledge by showing that it is the physical environment that matters most to British individuals but the social environment for Javanese. British people were more likely than Javanese people to make adaptations to their immediate environment, for example: downsizing after their children left, moving closer to nature, moving to a house with a garden, and generally creating spaces that were more age-friendly. In contrast, the Javanese were more focused on the importance of having good neighbors and living close to family. This phenomenon can be explained by the collectivist culture (Triandis, 2001) among the Javanese, which prioritizes family and community ties. Additionally, Soetjiningsih et al. (2019) stressed the importance of two crucial principles in the social and life patterns of Javanese society, *rukun* (harmony) and *urmat* (respect), which are both linked to one’s social environment. Furthermore, although both cultures recognized the significance of maintaining social relationships, older British individuals tended to place greater emphasis on the importance of friendships, whereas older Javanese individuals prioritized familial ties; consistent with previous findings from non-Western studies (Amin, 2017; Shiraz et al., 2020; Soetjiningsih et al., 2019).

Interestingly, some Javanese participants mentioned financial matters causing interpersonal conflict within their social network (presumably of increased importance due to the value they place on social relationships), especially with children and grandchildren. This may highlight the lack of financial security for some of the older people in Java, the existence of familial expectations regarding intergenerational financial support/filial piety, and the urgent need for social security/old age income/pensions for Javanese older adults. Similar findings were also found in a study of Singaporean older adults (Shiraz et al., 2020), which highlighted that discrepancies in financial expectations between older adults and their financial providers, often their children, were a major source of conflict. In our sample, although conflict was apparent, the overarching emphasis was on acceptance and harmony, consistent with the Javanese emphasis on maintaining relational and familial ties.

### Strengths and Limitations

This present study adds to our understanding of older people’s perspective across different cultures through qualitative research that directly captures people’s experiences on what it means to age well. Nevertheless, it is important to exercise caution when considering the findings, as our sample consisted of individuals who were self-selected, which may have resulted in an underrepresentation of ethnic minority groups, and an over-representation of participants who were interested in this area of research, or who believed they were aging well. It should be noted that the present study required participants to have access to the requisite technology and a quiet room. Most British participants had access to social media. Most Javanese participants did not have access to social media but heard about this study from their family/relatives/friends’ social media. Future studies could reduce these requirements to gather a broader sample. Moreover, around half of the participants in each cultural group held a higher educational degree. This could have impacted our findings, as more highly educated individuals are less prone to experiencing loneliness and boredom in later life (Bordone et al., 2020).

### Implications

The presence of both similarities and differences between our two groups of participants in terms of their views on what it means to age well suggests that policymakers and healthcare practitioners should incorporate the perspectives of older individuals from different cultures into their planning for initiatives related to aging well. This approach will facilitate the development of culturally sensitive interventions that promote aging well across different populations, which is particularly important in the UK due to its culturally diverse population. Our data suggest that the efficacy of interventions aimed at promoting aging well is contingent upon several key factors, including optimal physical health, a positive psychological outlook, financial security, a supportive environment, and engagement with spirituality and religiosity. Given the diverse viewpoints held by older individuals from different cultural backgrounds, it is crucial to exhibit adaptability when designing interventions. For example: interventions aimed at promoting quality of life and increasing well-being for Eastern countries/cultures ought to focus on creating an appropriate social environment; for instance, providing residence in close proximity to family members, and allowing individuals time for spirituality and religiosity. For Western countries/cultures, interventions should prioritize the provision of an optimal physical environment: one that has fewer stairs, is close to amenities, and has a garden or is close to nature.

In terms of implications for future research, it would be useful to undertake further studies using a more robust and heterogeneous sample to determine whether our findings are likely to generalize across other populations and cultures. It is important that future research considers the religious beliefs of the participants to examine potential similarities and differences, particularly between older persons who identify as Christian or Muslim, or follow other religions.

## Conclusion

Cross-culturally, similar factors appear to help older adults age well. These include health, a positive outlook, financial stability, a safe environment, and the opportunity for spiritual and religious activities. Some of these factors act as springboards and contributors to other factors. For example, being physically capable enables older adults to engage in fulfilling activities, and spirituality and religiosity help build a positive outlook on life. However, the emphasis on these factors varies according to culture. For British older adults, having a secure and comfortable environment is important; for Javanese older adults, being close to family and having a vibrant social environment is more of a priority. Older individuals in Java frequently live in extended families or are surrounded by family living close by, who may assist them with daily activities, whereas older individuals in Britain generally live in nuclear families, requiring home modifications for independent living. Understanding these important factors but recognizing cultural variations within them will be important for policy makers when devising strategies to support populations in aging well.

## Supplementary Material

gbae085_suppl_Supplementary_Material
