# Supplementary material for: What Does It Mean to “Age Well” Among British and Javanese Older Adults? A Cross-Cultural Qualitative Study
Source: J Gerontol B Psychol Sci Soc Sci. 2024 May 18;79(7):gbae085. doi: 10.1093/geronb/gbae085 (PMC11200188; doi:10.1093/geronb/gbae085)
Supplement: gbae085_suppl_Supplementary_Material [file gbae085_suppl_supplementary_material.docx]

***The Journals of Gerontology, Series B: Psychological Sciences and Social Sciences* Supplementary Material: Sulandari et al. What Does It Mean to ‘Age Well’ Among British and Javanese Older Adults? A Cross-Cultural Qualitative Study.**

**Supplementary Section A. Interview schedule**

1. What does ageing well mean to you?
2. How would you describe it?
3. What makes you enjoy being older?
4. Prompt – examples of activities (what do you get up to?)
5. Prompt – time with friends and family (do you have more/less time to spend with friends/family?)
6. Environment - where you live/surroundings
7. What kind of things make you being feel happy?
8. Prompt – examples of activities (what do you get up to?)
9. Prompt – time with friends and family (do you have more/less time to spend with friends/family?)
10. Environment - where you live/surroundings
11. How would you describe a person who appears to be ageing well?
12. Could you describe what characteristics/personality you think support this?
13. What would an average day look like for a person who is ageing well?
14. Where do they live?
15. Explore family/friends/neighbours’ relationships
16. Do you know someone who is ageing well? How would you describe them and what characteristics do they have? What do you think supports the idea that they are ageing well?
17. What things in general might stop someone from ageing well
18. Do you think you are ageing well?
19. If yes, what characteristics/personality do you think support this?
20. If no, why not? What is stopping you?
21. What are the three top things that help ageing well?

That brings us to the end of the questions I have for you, but do you have anything you would like to add or any questions for us? Thank you for sharing your thoughts with us.

**Supplementary Section B. Participant characteristics**

***Table S.1. Javanese Participants’ characteristics***

| **P** | **Sex** | **Age range** | **Educational background** | **Marital status** | **Living arrangement** | **Number of Health condition** | **Interview length** |
| --- | --- | --- | --- | --- | --- | --- | --- |
| J1 | F | 71-80 | Graduate Diploma | Widowed | Child and Grandchildren | 2 | 80’ |
| J2 | M | 61-70 | Junior High School | Married | Wife and youngest child | 0 | 30’ |
| J3 | F | 61-70 | Masters degree | Married | Husband | 1 | 36’ |
| J4 | M | 61-70 | Bachelors degree | Widowed | Youngest child | None | 55’ |
| J5 | F | 71-80 | Elementary school | Widowed | Youngest child | 2 | 38’ |
| J6 | F | 61-70 | Graduate Diploma | Widowed | Alone | 0 | 30’ |
| J7 | M | 61-70 | Bachelors degree | Married | Wife | 1 | 38’ |
| J8 | F | 61-70 | Bachelors degree | Married | Husband | 0 | 28’ |
| J9 | M | 71-80 | Senior high school | Married | Wife, grandchild | 0 | 47’ |
| J10 | F | 61-70 | Elementary school | Married | Husband; grandchild | 0 | 33’ |
| J11 | M | 61-70 | Bachelor s degree | Married | Wife, child | 1 | 35’ |
| J12 | F | 61-70 | Bachelors degree | Married | Husband, daughter’s family | 0 | 47’ |
| J13 | M | 61-70 | Senior high school | Widowed | Child and Grandchildren | 0 | 49’ |
| J14 | M | 71-80 | Bachelors degree | Widowed | Alone | 0 | 48’ |

Notes. F = Female; M = Male; P = Participant.

***Table S.2. British Participants’ characteristics***

| **P** | **Sex** | **Age range** | **Educational background** | **Marital status** | **Living arrangement** | **Number of Health Condition** | **Interview length** |
| --- | --- | --- | --- | --- | --- | --- | --- |
| B1 | F | 61-70 | Doctoral degree | Widowed | Alone | 0 | 23’ |
| B2 | F | 71-80 | High national diploma | Widowed | Alone | 1 | 35’ |
| B3 | F | 61-70 | Foundation background | Married | Husband and one child | 3 | 30’ |
| B4 | F | 71-80 | Bachelors degree | Divorce | Alone | 0 | 33’ |
| B5 | F | 61-70 | College 18 | Married | Husband | 2 | 46’ |
| B6 | F | 71-80 | Currently doing PhD | Single | Alone | 4 | 55’ |
| B7 | F | 61-70 | Honours degree | Widowed | alone | 0 | 50’ |
| B8 | M | 71-80 | O level | Married | Wife | 3 | 40’ |
| B9 | F | 61-70 | Bachelors degree | Married | Husband | 0 | 35’ |
| B10 | M | 61-70 | Bachelors degree | Married | Wife | 0 | 40’ |
| B11 | F | 71-80 | Diploma | Married | Husband | 2 | 43’ |
| B12 | M | 61-70 | Technical college; college of music | Married | Wife | 2 | 36’ |
| B13 | M | 71-80 | Masters degree | Married | Wife | 0 | 35’ |
| B14 | F | 61-70 | Masters degree | Married | Husband | 0 | 33’ |
| B15 | F | 61-70 | PVC teacher qualification | Widowed | Alone | 1 | 47’ |

Notes. F = Female; M = Male; P = Participant.

**Supplementary Section C. An example of disagreement between authors**

Whether the importance of “social factors” would stand alone as a theme or should be integrated with the theme of environment provides a useful example of author dispute and resolution when it came to coding the data. The earliest discussion of the environment theme referred to the physical environment, and did not include social aspects. After discussion among the authors and looking through the data again and considering the entire meaning, the authors decided to split the social factor into social activities and social connection and to merge social connection into the social environment factor and discuss this coding under the "having enough and feeling safe" theme. Social activities were reassigned to the “good health” theme since good health contributed to the ability of the older people to engage in social activities.

**Supplementary Section D. Themes, description, and example quote(s)**

***Table S.3. Themes, description, and example quote(s)***

| **Themes** | **Description** | **Example quote(s)** |
| --- | --- | --- |
| Good health facilitates the activities which lead to ageing well  B1, B2, B3, B4, B5, B6, B7, B8, B9, B10, B11, B12, B13, B14, B15  J1, J2, J3, J4, J5, J6, J7, J8, J9, J10, J11, J12, J13, J14 | Good health is the primary source for older people to engage in activities. The activities cover:   1. physical activities 2. social activities, 3. religious practices and activities 4. explore new interests and hobbies   It is not only physical health critical, but also mental and cognitive. The reason for OA to be healthy is also to be independent and not be a burden for anyone else. To keep healthy, older people were also being aware that they had to have a good lifestyle, such as: doing exercise, consuming healthy foods, avoiding smoking and alcohol, manage time between activity and rest. | - Keeping yourself fit and healthy is important because if you're full of ailments, you can't go out and meet people, and unfortunately, people get fed up with you moaning about your health. (B4) - If people are healthy, it means they can worship in peace, right? People will not be able to worship when they have health problems, so being healthy is important. Being healthy means being able to enjoy everything. For example, if I have an event or gathering invitation, I will be able to attend when I am healthy, but if I am sick, I will not be able to make it. Usually, after the *Fajr* prayer, I do recite, but yesterday I caught a cold, so it is not possible to recite the *Quran* for the following 2 to 3 days. So, with God’s willing, if you are healthy, you will definitely be able to do the religious practices. Being in good health also allows me to go for a walk in the morning. (J1) - When you are in good health, you can enjoy life. That is ageing well... It is still important to take care of your health… being able to take care of it through a proper lifestyle and a positive mindset, as well as having a balance between activities and rest. (J2) - Always keep being healthy by eating well, doing exercise, keeping busy, making sure that any ailments that come up are dealt with, and taking the appropriate food supplements. Going to the shops, surrounding area, or church, by walking. So, I do quite a lot of walking and Pilates. (B6) - I initially found the pandemic very difficult because I wasn't seeing friends and family face-to-face, but I've gotten used to it now. I've lost a bit of confidence about making a lot of face-to-face contact with people. I'm going to try and get that confidence back up again. So, I actually need to try and venture out more and meet people face-to-face more. (B5) - If you have to rely on other people to come and pick you up, all the rest of it becomes a bit of a pain for people. I don't like to bother people, so it's best to try and keep healthy. (B7) - I thank God. I was accompanied by health from *Allah*, so I don't bother my children, grandchildren, or neighbours. I think that's good. (J6) - If people become depressed, it's difficult to get motivated to do things. (B2) - I think if you're ageing well, mentally bright, and able to communicate, then that's a nice thing to be able to do that a lot of people can't do most of the time because of dementia. I find that a lot of people forget things and don't behave the way they would normally. If I had a mental health issue or dementia, then there's no way that I could work on my films and still edit them and be creative. I do a lot of writing as well, so I'm very pleased that my brain still functions normally. (B8) - Try to keep your mind active by doing number puzzles and word puzzles. A lot of my friends do jigsaw puzzles and things like that. It is important to keep your mind active, so do some sort of thing like that or quizzes, even if you watch them on television, and try to answer the questions, it keeps your intellect going. (B7) |
| Holding a positive outlook is a decisive factor in ageing well  B1, B2, B3, B4, B5, B6, B7, B8, B9, B10, B11, B12, B13, B14, B15  J1, J2, J3, J4, J5, J6, J7, J8, J9, J10, J11, J12, J13, J14 | Viewing life positively helps to accept any circumstances and leads to which lead to enjoy life, being satisfied with life, being happy, and being content.  This theme also identified how the personality of ageing well in both cultures. | - People who are ageing well are those who are more positive and are not denying the difficulties they've had in their lives. They tend to have a more positive attitude, not being judgmental of other age groups or ethnicities, accepting other people, and not complaining all the time. (B5) - Ageing well is an optimistic attitude; keep going, don't give up, don't get tired, and keep the spirit up. (J12) - Yeah, personality helps to age well. If we're not friendly and don’t communicate with people, it's difficult. You know, to get that conversation going, some people are not quite as easy to talk to, and it can be quite difficult. (B2) - as you get older, you become more confident in your ability to do things, which is good. Ageing well means to always be cheerful, sociable, kind, and helpful, and have a sense of humour. [Also, it helps to be] determined, and if you decide to do something then do it, and if a problem gets in the way, defeat it. (B4) - The benefits [of having positive outlook] are that you never feel like you "have nothing to do," meaning you're not really lonely. So, life is good, but there are still hopes. (J14) - I would say ageing well means being outgoing, being the sort of open and friendly person without bringing their own problems to any relationship, and being good at communicating. (B12) - *Allah* teaches us to be patient. You have to be patient and *tawakkal* (put all of your effort into doing something, but then again, be patient). So that we can have peace and no trouble in our lives. Right, if you are patient, there will be no problem. (J9) - People who are ageing well are very kind and thoughtful. I'm very friendly and can talk [nicely] to people. So, I think that helps me age well. (B2) - Being patient [helps me] to avoid conflict and understand others' circumstances. (J10) - She's always had a positive attitude towards things. She'll tackle modern things; she doesn't say, oh, I can't work on my computer, I'm too old. Those who are ageing are well prepared to look at the positive in the world and keep smiling. (B4) |
| "Having enough" and "feeling safe" provide peace of mind  B1, B2, B3, B4, B5, B6, B7, B8, B9, B10, B11, B12, B13, B14, B15  J1, J2, J3, J4, J5, J7, J8, J9, J10, J11, J12, J13, J14 | Finance and the environment were necessary for older people. This theme explains how the older Javanese "have enough" regarding finance and the living environment. While both cultures mentioned the importance of physical and social environment, the older British highlighted more on physical environment, and the Javanese put more stress on the social environment. | - So, then my condition like this is more than enough; it's more than enough because I don't look up, I look down. Ageing well is not the one above, right? Just as it is. (J1) - From my point of view, ageing well for older people means feeling like you have enough. For example, my house is just simple, but I feel that’s enough, and I am grateful. (J5) - If they have financial problems, I think someone is going to be worried all the time about money; at the moment, they can't imagine how they're going to cope with their heating bills. That will make people anxious; it will affect their mental health and things like that. (B9) - I am fortunate. Fortunately, I don't have to worry about my day-to-day finance. (B15) - Economic conditions also affect relationships. What makes the family relationship not good is usually economic problems. There are neighbours of mine who have bad relationships with their neighbours because of economic problems. (J8) - It's simply an economic problem. If economic stability isn't there, some people tend to get upset easily, argue with their children and grandchildren, and so on. (J10) - Well, I think they (who are ageing well), live at home and not in a care home. (B8) - A nursing home is a place to stay when you are not very well. So, a person who is ageing well is very independent. Even though they might not be physically well, they will try to find ways to live their lives independently. (B15) - All my three children are already married. One of them lives a bit far away with her husband, but my two sons live either side of me: my house is right in the middle. In accordance with what I dreamt of, I feel comfortable in my old age because my children and grandchildren are around me. (J2) - We don't have family close by. [But it is helpful to be] with friends and have a nice chat together and talk about something interesting; talk about problems. If they've got some difficulty, they feel they're able to ask for help. (B14) - I keep in touch with my friends by, for example, having lunch with the girls and occasionally going out. So, I mostly go for dinner; but sometimes one or two friends will invite me to stay in a house. (B15) |
| Spirituality and religiosity provide tranquillity  B3, B5, B11, B12, B13, B14  J1, J2, J3, J4, J5, J6, J7, J8, J9, J10, J11, J12, J13, J14 | This theme explored how faith or religious belief drove the participants' viewpoints. Both spirituality and religiosity provide tranquillity in the older participants' life. Belief about the afterlife also motivated them to do good deeds and practice religious commands to obtain enough provision in eternal life. | - I do recite in a religious gathering, sometimes praying and reciting. I learned the *Quran's* translation when I was a child. I learned to recite when I was a child. So, I do know how to recite. I also worship in the mosque. If you hear the call to prayer, you immediately pray. (J5) - The problem is that it's very important that, the older we get, the closer we get to returning to *Allah.* That means we have to have a lot of provisions. (J1) - Religious practices are conducted to provide for the afterlife. The next day, when you leave this world as you are getting older, what you're looking for is having enough provision in the afterlife so that *Allah* will accept it and grant us a heaven. (J9) - I think it's a Christian outlook. It's a spiritual and faith thing that supports my ability to enjoy life and to be content. In terms of our attendance at church and our reading and prayer, that is key to the way we are, I feel. The way that God has been faithful to us and given us the life that we have at the moment. (B12) - Praying is good. It turns out that the reward that can already be felt is being tranquil. Practicing the night prayer, the dhikr, and getting close to *Allah* is very good. Talking to *Allah* makes me feel very good. (J14) - I can read the *Quran*; something happened when, after prayer, I read it. It made my heart calm. (J10) - Reading the Quran provides peace of mind; it turns out to be true. I often read the Quran and feel satisfied and happy. (J12) - The ageing process is just a natural part of life. I pray, meditate, and talk for God. It's that sense that there is something much greater than me. There is a greater power that gives me a sense of meaning. [B5] |

**Supplementary Section E. Figures for the proportion of codes.**

Codes are compared by the proportion of the number of a) participants coded and b) quotes coded.


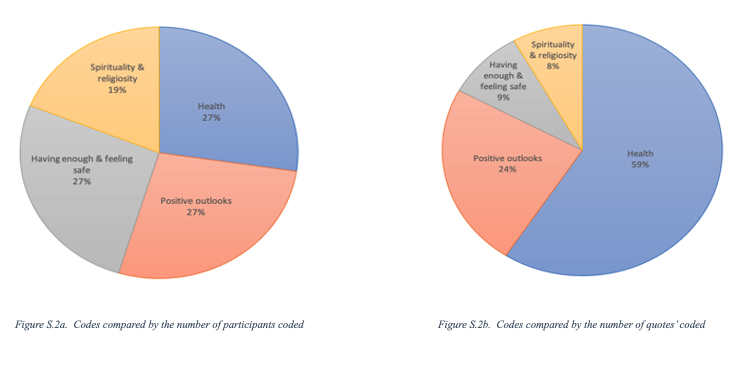


**Supplementary F. Diagrams of participants distribution based on the codes.**

Figure S.3a. The diagram of participants distribution on the importance of psychological and cognitive health.

Figure S.3b. The diagram of participants distribution on the importance of being patient and outgoing.

Figure S.3c. The diagram of participants distribution on the physical and social environment
